# Rationale and design of the BeyeOMARKER study: prospective evaluation of blood- and eye-based biomarkers for early detection of Alzheimer’s disease pathology in the eye clinic

**DOI:** 10.1186/s13195-024-01545-1

**Published:** 2024-08-21

**Authors:** Ilse Bader, Colin Groot, H. Stevie Tan, Jean-Marie A. Milongo, Jurre den Haan, Inge M. W. Verberk, Keir Yong, Julie Orellina, Shannon Campbell, David Wilson, Argonde C. van Harten, Pauline H. B. Kok, Wiesje M. van der Flier, Yolande A. L. Pijnenburg, Frederik Barkhof, Elsmarieke van de Giessen, Charlotte E. Teunissen, Femke H. Bouwman, Rik Ossenkoppele

**Affiliations:** 1 Amsterdam Neuroscience, Neurodegeneration, Amsterdam, 1081 HV The Netherlands; 2 Alzheimer Center Amsterdam, Neurology, Vrije Universiteit Amsterdam, Amsterdam UMC location VUmc, Amsterdam, 1081 HZ The Netherlands; 3 Department of Ophthalmology, Bergman Clinics, Amsterdam, 1101 BM The Netherlands; 4 Department of Ophthalmology, Amsterdam UMC, Amsterdam, 1081 HV The Netherlands; 5 Amsterdam Neuroscience, Brain Imaging, Vrije Universiteit Amsterdam, Amsterdam, 1081 HV The Netherlands; 6 Amsterdam UMC Location VUmc, Amsterdam Reproduction and Development Research Institute, Vrije Universiteit Amsterdam, Amsterdam, 1081 HV The Netherlands; 7 Neurochemistry Laboratory, Laboratory Medicine, Amsterdam Neuroscience, Vrije Universiteit Amsterdam, Amsterdam UMC Location VUmc, Amsterdam, 1081 HV The Netherlands; 8 Queen Square Institute of Neurology, Dementia Research Centre, London, WC1N 3BG UK; 9 Optina Diagnostics, Montréal, QC Canada; 10 Quanterix Corporation, Billerica, MA USA; 11 Epidemiology and Data Science, Vrije Universiteit Amsterdam, Amsterdam UMC Location VUmc, Amsterdam, 1081 HV The Netherlands; 12 Radiology & Nuclear Medicine, Vrije Universiteit Amsterdam, Amsterdam UMC Location VUmc, Amsterdam, 1081 HZ The Netherlands; 13 UCL Queen Square Institute of Neurology and Centre for Medical Image Computing, University College, London, WC1N 3BG UK; 14 Clinical Memory Research Unit, Lund University, Lund, Sweden

**Keywords:** Alzheimer’s disease, Screening, Blood-based biomarkers, Plasma p-tau, Hyperspectral retinal imaging, Retinal imaging, Eye clinic, Visual impairment, Age-related eye disorder

## Abstract

**Background:**

Alzheimer’s disease (AD) is a common, complex and multifactorial disease that may require screening across multiple routes of referral to enable early detection and subsequent future implementation of tailored interventions. Blood- and eye-based biomarkers show promise as low-cost, scalable and patient-friendly tools for early AD detection given their ability to provide information on AD pathophysiological changes and manifestations in the retina, respectively. Eye clinics provide an intriguing real-world proof-of-concept setting to evaluate the performance of these potential AD screening tools given the intricate connections between the eye and brain, presumed enrichment for AD pathology in the aging population with eye disorders, and the potential for an accelerated diagnostic pathway for under-recognized patient groups.

**Methods:**

The BeyeOMARKER study is a prospective, observational, longitudinal cohort study aiming to include individuals visiting an eye-clinic. Inclusion criteria entail being ≥ 50 years old and having no prior dementia diagnosis. Excluded eye-conditions include traumatic insults, superficial inflammation, and conditions in surrounding structures of the eye that are not engaged in vision. The BeyeOMARKER cohort (*n* = 700) will undergo blood collection to assess plasma p-tau217 levels and a brief cognitive screening at the eye clinic. All participants will subsequently be invited for annual longitudinal follow-up including remotely administered cognitive screening and questionnaires. The BeyeOMARKER + cohort (*n* = 150), consisting of 100 plasma p-tau217 positive participants and 50 matched negative controls selected from the BeyeOMARKER cohort, will additionally undergo Aβ-PET and tau-PET, MRI, retinal imaging including hyperspectral imaging (primary), widefield imaging, optical coherence tomography (OCT) and OCT-Angiography (secondary), and cognitive and cortical vision assessments.

**Results:**

We aim to implement the current protocol between April 2024 until March 2027. Primary outcomes include the performance of plasma p-tau217 and hyperspectral retinal imaging to detect AD pathology (using Aβ- and tau-PET visual read as reference standard) and to detect cognitive decline. Initial follow-up is ~ 2 years but may be extended with additional funding.

**Conclusions:**

We envision that the BeyeOMARKER study will demonstrate the feasibility of early AD detection based on blood- and eye-based biomarkers in alternative screening settings, and will improve our understanding of the eye-brain connection.

**Trial registration:**

The BeyeOMARKER study (Eudamed CIV ID: CIV-NL-23–09-044086; registration date: 19th of March 2024) is approved by the ethical review board of the Amsterdam UMC.

**Supplementary Information:**

The online version contains supplementary material available at 10.1186/s13195-024-01545-1.

## Background

The hallmark pathophysiological processes of Alzheimer’s disease (AD; i.e., amyloid β [Aβ] plaques and neurofibrillary tau tangles) may emerge 20–30 years prior to the onset of dementia, and the earliest incipient symptoms often go unnoticed by patients and their caregivers [1–3]. Early AD, prior to extensive atrophy and cognitive impairment, is the optimal window for intervention and may be essential to achieve the most beneficial long-term outcomes [4–6]. This notion has led to a paradigm shift towards a focus on early biomarker-confirmed diagnosis and biological staging of AD [1], which is further fueled by the first regulatory approvals of monoclonal antibodies against Aβ [7, 8] and by clinical trial results that have hinted towards more beneficial outcomes in the early, pre-symptomatic stages of AD [9]. These developments are major advances in the field but also emphasize longstanding challenges concerning the rising demand for large-scale accessibility of early AD detection to facilitate early intervention [10]. The current diagnostic process in memory clinics is inadequate to accommodate large-scale early detection of AD pathology due to the reliance on expensive and invasive procedures (i.e., a lumbar puncture or Positron Emission Tomography [PET]) [1, 3]. Furthermore, PET and cerebrospinal fluid (CSF) biomarkers are only clinically approved (e.g., European Commission [CE-marked] or US Food and Drug Administration [FDA] approved) to diagnose individuals at symptomatic stages of AD and are only accessible in highly specialized clinics that are mainly situated in high-income countries. To prepare for a future wherein disease-modifying treatment may become widely available, there is a need towards building an efficient and inclusive infrastructure to detect individuals at risk of AD. This will require low-cost, patient-friendly and scalable biomarkers for AD that are also suitable for implementation outside of a specialized memory clinic setting, such as blood-based and eye-based biomarkers [11]. Blood-based biomarkers for AD have advanced rapidly and hold promise for future real-world clinical implementation to detect AD pathophysiology [12, 13]. Eye-based biomarkers derived from retinal imaging are emerging to screen for AD-associated structural changes and Aβ- or tau-related lesions, which may be of particular relevance in ophthalmological settings [14–16]. The BeyeOMARKER study aims to evaluate the real-world implementation of blood-based biomarkers, and the potential (additional) value of eye-based biomarkers, to screen for AD pathophysiology in eye-clinics. In this design paper, we provide a rationale for early detection of AD in eye clinics, present the BeyeOMARKER study design and population, and elaborate on several aspects of the study including ethical considerations, potential challenges, and future opportunities.

### Rationale

Based on previous epidemiological and pathophysiological evidence, eye clinics provide a prime opportunity to investigate the feasibility of blood- and eye-based biomarkers to detect early AD. From an epidemiological perspective, eye clinics are known for a high-throughput of patients within the typical age-range when AD pathological changes first manifest, highlighted by the overlap in age-of-onset (i.e., > 50 years of age) for acquired eye-disorders [17–19] and AD [1, 20–26]. Moreover, epidemiological investigations indicate that eye patients may be at increased risk for dementia and AD [27–35] (Table 1). These associations are reported for glaucoma, age-related macular degeneration, diabetic retinopathy, cataract, and for vision impairment as a whole. Possible mechanisms underlying this increased risk may differ per eye condition, and could be related to embryological, anatomical, physiological and functional connections between the eyes and the brain. Through these intricate connections, diseases affecting the brain may affect the eye and *vice versa* [36, 37]. Indeed, ocular manifestations of AD are myriad and include the retinal presence of AD pathology, neurodegenerative changes and vascular changes [15, 16, 37–41] (Table 2). Various hypotheses have been postulated to explain the association between eye disorders and AD, such as shared (genetic) risk factors, the common-cause hypothesis, or the sensory deprivation and information degradation hypotheses [29, 34, 42–48] (Table 3). For example, glaucoma and age-related macular degeneration are neurodegenerative diseases of the eye that share pathological features with AD, such as the presence of Aβ- and tau deposits and inflammatory and neurodegenerative processes [49–51]. For cataract on the other hand, alternative reversible cognitive or psychosocial processes may be involved given that cataract extraction appears to reverse dementia risk [52, 53]. Taking together these close connections between the eyes and the brain, the eye is considered an accessible ‘window to the brain’ and eye-based biomarkers have potential as a prognostic tool to identify risk of cognitive impairment due to neurodegenerative disease [15, 36, 37, 39]. Moreover, vison impairment represents an established modifiable risk factor (population attributable fraction 1.8% [54]) and early and effective treatment of eye disorder may hence lower the odds of developing dementia [55, 54, 56].

**Table 1:** Epidemiological eye-brain connections

Epidemiological eye-brain connections
Accumulating evidence suggests an association between eye diseases and visual impairment with (AD) dementia. Results for specific eye disease are mixed and effect sizes vary considerably, possibly due to different definitions for eye diseases and visual impairment (e.g. subjective and objective), different criteria for AD (e.g. not always biomarker-confirmed) and cohort differences (e.g. age and presence of comorbidities). Reported association include:
• **Co-existence of eye disease and cognitive impairment:** In a systematic review and meta-analysis across 57 studies, the estimated co-existence between eye disease and cognitive impairment varied but was estimated to be 8.4–52.4%, 12.3–90.2% and 3.9–77.8% for AMD, glaucoma and DR patients, respectively [27].
• **Eye disease as risk factor**: In a first meta-analysis, increased risk on AD was reported for DR (HR = 1.29 [95%CI: 1.03–1.61]) and cataract (HR = 1.26, [1.07–1.48]) but not for AMD and glaucoma [28]. In contrast, subsequent meta-analyses did report associations for AMD and glaucoma. The first reported an association between AMD and AD (HR = 1.21 [1.03–1.43]) and observed that the association was stronger in dry AMD than wet AMD [172]. The second reported an association between AD and glaucoma (HR = 1.39 [1.35–1.43]) particularly at older age, which applied specifically to primary open-angle glaucoma (HR = 1.31 [1.27–1.36]) and normal-tension glaucoma (HR = 1.28 [1.20–1.36]), but not primary narrow angle glaucoma [173].
• **Visual impairment as risk factor**: Visual impairment has been consistently associated with an increased risk on dementia [34] and this association appears stronger with increasing levels of visual impairment severity [29, 30]. For specifically AD, an association for at least mild visual impairment compared to no visual impairment (RR = 1.47 [95%CI: 1.43–1.50]) has been reported across two studies but this evidence is more sparse [28].

*Abbreviations*: *AD* Alzheimer’s disease, *AMD* Age-related macular degeneration, *DR* Diabetic retinopathy, *HR* Hazard ratio, *RR* Risk ratio, *CI* Confidence interval

**Table 2:** Pathophysiological eye-brain connections

Pathophysiological eye-brain connections
The eyes are described as ‘window to the brain’ based on many commonalities:
• **Embryological:** The retina and the brain both originate from the diencephalon during embryonic development and remain structurally and functionally connected throughout life.
• **Anatomical**: Both the retina and the brain are characterized by presence of a layered cytological structure, containing similarly structured neurons and axons, and a similar (micro)vascular structure that includes presence of a blood-retina/brain barrier,
• **Physiological:** The retina and the brain share multiple physiological processes, including neural processing, myelination by oligodendrocytes, and degenerative and regenerative processes.
Based on these commonalities, diseases affecting the brain can be expected to affect the eye and *vice versa* [36]. Indeed, ocular manifestations of AD are myriad and include retinal presence of AD pathology, neurodegeneration and changes in vasculature. Retinal AD pathology includes presence of amyloid peptides and plaques, vascular amyloid depositions, and tau pathology [42, 174, 175] which correlate with AD pathology burden in the brain and general cognition [42, 175]. For retinal neurodegenerative and vascular changes, the most extensively reviewed parameters are derived from OCT (e.g. retinal thinning and loss of retinal ganglion cells) and OCT-A (e.g. vessel density and tortuosity). These parameters generally differ between AD patients versus controls and correlate with cognition [14–16, 37–39]. Though the discriminative specificity for AD for single OCT and OCT-A parameters is debated, the retina can be imaged using a diverse array of non-invasive techniques, thereby providing access to a wide range of biomarkers that can serve as biomarkers to predict pathology in the brain.

*Abbreviations*: *AD* Alzheimer’s disease, *OCT* Optical Coherence Tomography, *OCT-A* OCT-Angiography

**Table 3:** Hypothesized explanations for the eye-brain connection

Hypothesized explanations for the eye-brain connection
The exact mechanisms linking eye disease and sensory deficits with Alzheimer’s disease are yet to be elucidated, but could involve (a combination of) the following hypotheses [47, 48, 58, 59, 176]:
• **Common cause hypothesis:** Both visual and cognitive impairment are a result of a shared (possibly age-related, vascular or inflammatory) pathological mechanisms that affect both the eyes and the brain.
• **Shared risk factors:** Risk factors including age, smoking, diabetes, obesity, lower socio-economic status and vascular risk factors could (independently) lead to both eye and brain disorders.
• **Sensory deprivation hypothesis:** visual impairment leads to reduced visual stimulation of cortical vision areas, resulting in atrophy and reorganization. These physical changes in turn affect cognitive processing and performance.
• **Information degradation hypothesis:** Impaired vision leads to degraded visual input which results in perceptual processing errors and increases the cognitive load required to adequately perform visual tasks. As more cognitive resources are allocated to perception, higher-order cognitive processes may be compromised.
• **Consequences of visual impairment are a risk factor:** The connection between visual impairment and cognition could be mediated by an association between visual impairment and social isolation, decreased physical activity and depression.
• **Detection bias:** Alternatively, use of vision-dependent neuropsychological testing could lead to underperformance in individuals who have a visual impairment, leading to cognitive test bias affecting the relation between visual impairment and cognitive impairment.

Another highly relevant factor contributing to the suitability of eye clinics as a screening setting for AD is related to the potential for an accelerated diagnostic pathway for currently under-recognized or underserved patient groups. First, individuals with an eye disorder represent a large portion of the aging population (e.g. prevalence of mild and moderate/severe visual impairment in individuals ≥ 50 years is estimated to be 7.7% and 11.2%, respectively [57]), and they appear to be disproportionately affected by AD [28–31]. This group experiences particular diagnostic challenges and underrepresentation in clinical research and trials due to accessibility issues (e.g., difficulties in traveling) and confounding of visually-mediated neuropsychological assessment [58–62]. Second, individuals with a low income, relatively low education attainment and a minority status are known to be disproportionally affected by AD [63–65]. These individuals typically experience difficulties in cognitive testing due to cultural bias and/or language barriers [66] and are currently underrepresented in memory clinic populations [67] and in clinical trial samples [62, 68]. Eye clinics provide an alternative route to connect with individuals who are otherwise unlikely to seek help if they experience cognitive complaints, for example due to dementia-related stigma or lack of awareness in some diverse communities [69]. Third, individuals with an atypical clinical presentation of AD generally experience significant morbidity and impact on daily life, but are diagnosed relatively late due to their atypical (non-amnestic) clinical presentation and overrepresentation in younger-onset AD [70–72]. Of particular interest in the eye clinic are individuals suffering from posterior cortical atrophy (PCA), also referred to as the visual-variant AD. PCA is characterized by early and prominent impairment in visual perception or visuospatial processing accompanied by pathology and atrophy that disproportionally affects the visual and visual association cortices [73, 74]. These individuals may present at the eye clinic due to their visual impairments but, as the cause is rooted in the brain rather than the eye, the complaints often remain unexplained by an ophthalmologist [72, 75, 76]. These factors may contribute to the long interval of on average 3.8 years between symptom onset and a formal PCA diagnosis [74]. Shortening this interval is essential to provide these patients with more equal access to patient management and to move towards clinical trial opportunities [75]. For all of the aforementioned individuals, eye clinics may provide an accelerated diagnostic pathway where the use of a biological (rather than cognitive) marker for AD could mitigate cognitive test(ing) bias, and the use of patient-friendly tools may reduce barriers to participation in research [77, 78]. By exploring the potential for AD detection in diverse and alternative setting, the BeyeOMARKER study aims to contribute to a more inclusive healthcare system.

### Screening biomarkers in the BeyeOMARKER study

The main biomarkers of interest for the BeyeOMARKER study are the blood-based plasma phosphorylated tau (p-tau217) biomarker and eye-based hyperspectral (HS) retinal scans.

#### Blood-biomarker measurement: plasma p-tau217

Blood-based biomarkers have seen a rapid rise to prominence as minimally invasive tools to detect AD pathology [12]. Emerging blood-based biomarkers for AD include markers for the hallmark pathologies (p-tau isoforms and Aβ) and markers of axonal degeneration (neurofilament light; NfL) or astrocytosis (glial fibrillary acidic protein; GFAP [12]). Since future high-throughput analysis of blood-based AD biomarkers will require the use of standardized and commercially available assays [12], we will screen participants based on the commercially available Quanterix single-molecule array (Simoa) for plasma p-tau217. Several p-tau isoforms exhibit high analytical and clinical performance [79–86], are specific to AD [87], and have adequate predictive value for atrophy and cognitive measures [82, 83, 88–90]. However, p-tau217 appears to be most accurate in detecting the earliest AD pathological changes [91–95] and correlates strongly with postmortem Aβ plaques and tau tangle load [93].

#### Eye-based screening: hyperspectral retinal scanning

Eye-based biomarkers have gained attention over the years within the field of neurodegenerative diseases since the retina shares many characteristics with the brain [96] (Table 2). Moreover, it is the only part of the central nervous system that is not shielded by bone which makes non-invasive and high-resolution imaging relatively easy. In the BeyeOMARKER study, a subset of participants will undergo retinal scanning including a HS retinal scan developed by Optina Diagnostics (Canada). Standard retinal imaging techniques provide spatial information and have been used to show vascular and neurodegenerative changes in AD [14–16, 37–39]. HS retinal imaging additionally incorporates reflective properties of the retina in response to monochromatic light waves, and thereby produces retinal images containing both spectral and spatial information [97]. Retinal spectral differences (i.e., differences in reflection in response to certain wavelengths) have been detected between control and AD mouse models that accumulate amyloid, both *in vivo* [98, 99] and *ex vivo* [100, 101]. The data-rich retinal images provided by the HS retinal scan were used to train an artificial intelligence (AI) algorithm to detect retinal features associated with AD. This AI paradigm has demonstrated good discriminative ability between amyloid negative and amyloid positive individuals [97, 98, 102–104], as well as between clinically diagnosed AD cases versus cognitively unimpaired participants [105]. These earlier preliminary findings using HS retinal imaging highlight the potential of this biomarker in a prospective screening setting.

### Knowledge gaps

Despite the promising performance of blood- and eye-based biomarkers for AD, several aspects remain to be evaluated to ascertain their (potentially complementary) utility as early AD screening tools outside specialized memory clinics. First, clinical performance studies on blood-based biomarkers to date have included relatively homogeneous samples with high diagnostic certainty, were mostly retrospective in design, and did not use a priori defined cut-offs [13]. These study design aspects could have favored biomarker performance and hamper generalizability to many real-world clinical settings. Similarly, validation studies of HS retinal imaging against Aβ-PET have only been performed in selected populations without eye conditions and with a high diagnostic certainty for AD [15, 97, 98, 102–104, 106]. Secondly, the clinical value of blood- and eye-based biomarkers has been studied separately but they have not yet been examined as potentially complementary markers in a combined prediction model. We hypothesize that combining these biomarkers into an integrative or step-wise model will provide complementary or even additive diagnostic and prognostic value for AD since plasma p-tau217 allows highly specific detection of a hallmark of AD pathology whereas the (HS) retinal scans also allow minimally invasive visualization of a multitude of neurodegenerative, inflammatory, vascular, and AD-related pathological changes that are reflective of changes in the brain [10, 13, 14, 107]. Of note, the efficacy of AD screening in an eye clinic population also partially relies on whether this population is indeed enriched for AD pathology. Although individuals with an eye disorder are at increased risk for (AD) dementia [28–34], risk estimates vary, and a precise prevalence estimate for AD biomarker positivity within the eye clinic population is currently lacking.

### Study objectives

**Fig. 1:**
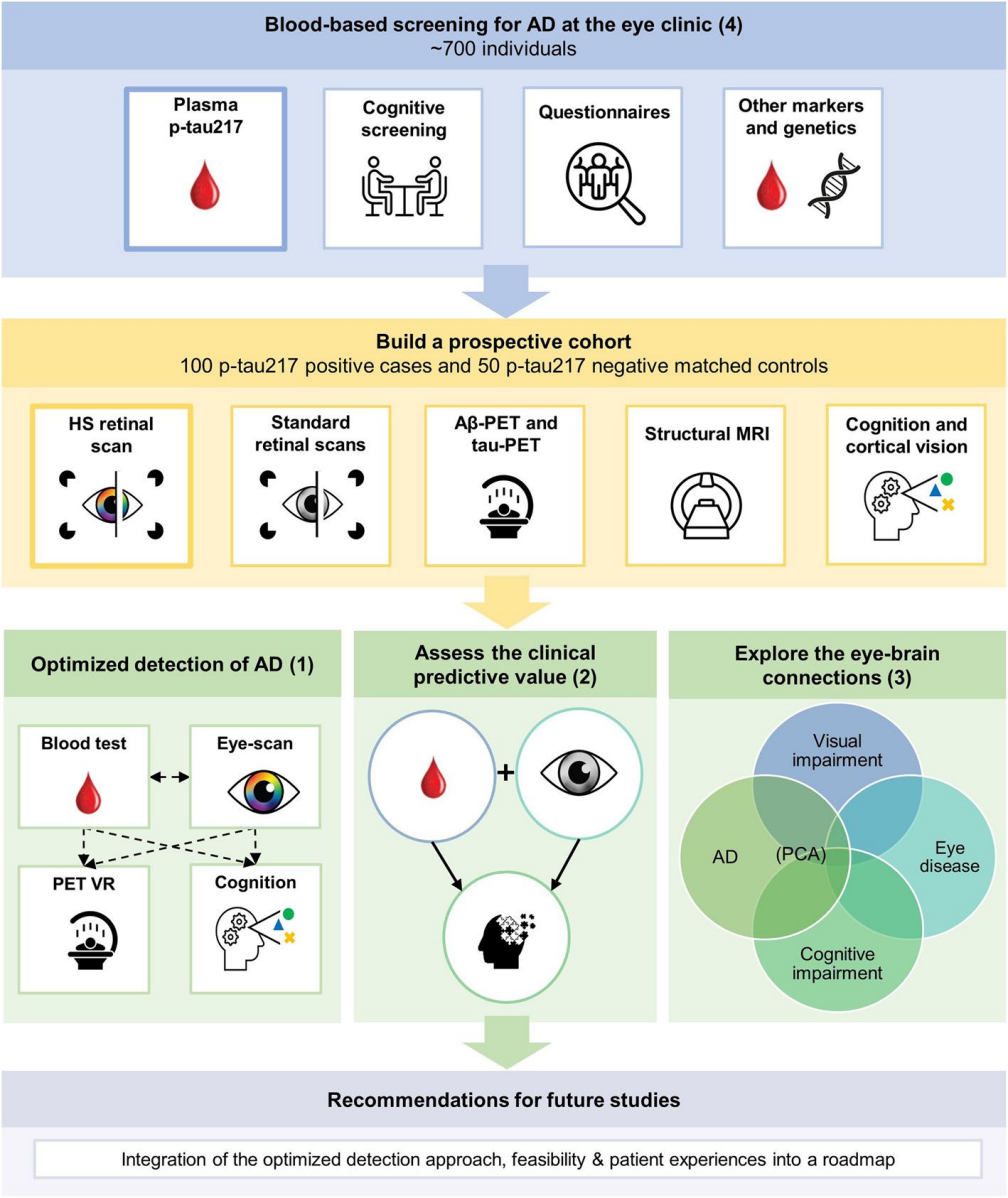
BeyeOMARKER study aims. *Abbreviations*: AD = Alzheimer’s Disease, PET = Position Emission Tomography, MRI = magnetic resonance imaging, Aβ = Amyloid beta, PCA = Posterior Cortical Atrophy

The primary aim of the BeyeOMARKER study is to evaluate and compare the performance of plasma p-tau217 and HS retinal scans to predict AD pathophysiology and cognitive decline (1). In addition, we envision that the BeyeOMARKER will provide a multimodal dataset for a diverse sample of patients visiting the eye clinic to secondarily (2) assess the individual and complementary clinical predictive value of other blood- and eye-based biomarkers, (3) explore the potential mechanisms contributing to the link between AD and conditions in the visual system, and (4) investigate enrichment for AD in an eye clinic population. The specific aims and their corresponding endpoints are also listed in Table 4 and visualized in Fig. 1. Findings of the BeyeOMARKER could ultimately aid in providing a roadmap for future studies on minimally invasive early detection of AD in alternative diagnostic settings.

**Table 4:** Objectives and endpoints of the BeyeOMARKER study

	**Objectives of the BeyeOMARKER study**	**Corresponding endpoints**
**Primary**	**1.** **Evaluate and compare the performance of plasma p-tau217 and HS retinal scans to predict AD pathophysiology and cognitive decline**	Accuracy of plasma p-tau217 and HS retinal scanning to detect 1) AD pathophysiology based on Aβ-PET and tau-PET visual read, and 2) clinical decline based on cognition (mPACC5)
**Secondary**	**2.** **Assess the individual and complementary clinical predictive value of other blood- and eye-based biomarkers**	Associations between blood-based biomarkers and eye-based biomarkers with down-stream effects of AD (e.g. atrophy, cognition and cortical vision)
	**3**. **Explore the potential mechanisms contributing to the link between AD and the conditions of the visual system**	Group-comparisons of neurobiological and cognitive manifestations in the BeyeOMARKER cohort versus a traditional memory clinic cohort, including suspected and confirmed PCA patients
	**4. Investigate enrichment for AD in an eye clinic population**	The observed prevalence of AD biomarker positivity in an eye clinic population compared to a modeled prevalence estimate for the general population

*Abbreviations*: *p-tau* Phosphorylated tau, *HS* Hyperspectral, *mPACC5* Modified preclinical Alzheimer cognitive composite 5, *AD* Alzheimer’s Disease, *Aβ* Amyloid beta, *PET* Positron Emission Tomography, *PCA* Posterior Cortical Atrophy

## Methods

### Study design

**Fig. 2:**
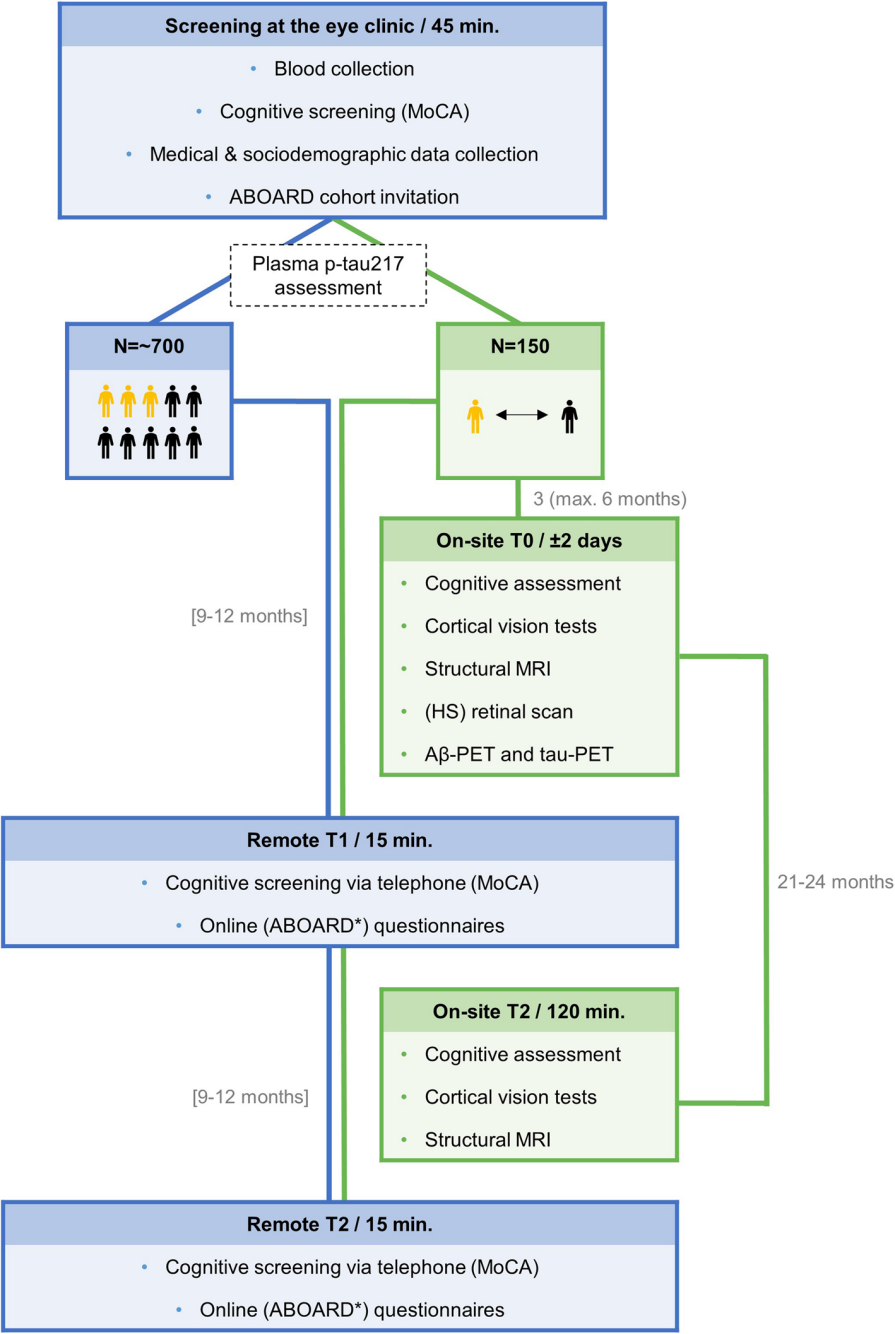
Study design including study visits, study procedures, time-intervals and the study population for all participants (blue route) and the BeyeOMARKER + cohort (green route). *Abbreviations*: MoCA = Montreal Cognitive Assessment, MRI = magnetic resonance imaging, HS = hyperspectral, ABOARD = A Personalized Medicine Approach for Alzheimer's Disease cohort study, yellow and black individuals represent the estimated plasma p-tau217 positive and negative individuals, respectively. *only applicable if the required optional consent has been provided

The BeyeOMARKER study is a single-center prospective, observational, longitudinal cohort study aiming to include individuals from a clinic for comprehensive eye-care who have no prior dementia diagnosis and are ≥ 50 years of age. As illustrated in Fig. 2, the BeyeOMARKER study comprises an initial screening phase, including the plasma p-tau217 assessment, followed by two longitudinal arms for subsequent follow-up. All BeyeOMARKER participants (prospected *n* = 700) will be followed-up remotely at T1 (9–12 months after screening) and T2 (9–12 months after T1). This will include online questionnaires and a web-based cognitive test (cCOG; [108]) partly in collaboration with the online ABOARD (“A Personalized Medicine Approach for Alzheimer's Disease”) platform [109], and cognitive screening via telephone (Fig. 2, blue route). In addition, from the full BeyeOMARKER cohort a BeyeOMARKER + subcohort will be recruited, which will consist of 100 plasma p-tau217 positive individuals and 50 plasma p-tau217 negative individuals matched on age, sex and eye condition. The BeyeOMARKER + cohort (*n* = 150) will be invited to the Amsterdam UMC for assessment at T0 (± 3 months and maximum 6 months after screening) and T2 (21–24 months after T0). Assessment at T0 includes standard and HS retinal imaging, structural MRI, Aβ-PET, tau-PET, and a cognitive and cortical vision test battery. Assessment at T2 includes a follow-up MRI and cognitive and cortical vision assessment (Fig. 2, green route). Outcomes available for the BeyeOMARKER and BeyeOMARKER + cohort are listed in Table S2 and will be described in further detail below. Additional funding will be sought to allow extended follow-up and repeated assessments.

### Targeted sample size

A conservative estimate of the prevalence of plasma p-tau217 positivity in cognitively unimpaired subjects between 65 to 69 years of age is 17.0% based on the lower bound of the 95%-confidence interval derived from a large meta-analysis on amyloid abnormality across the AD spectrum [110]. We estimated the plasma p-tau217 prevalence based on amyloid-based estimates since the two are strongly related to each other [111]. Based on an open access sample size calculator for prevalence studies [112], we subsequently estimated a required screening sample size of *n* = 700 (prevalence = 17.0%, level of confidence (Z) = 95%, precision estimate (D) = 3.0%, expected attrition rate = 10%). Given the expected prevalence of amyloid positivity (i.e., 17.0%), the screening sample of 700 subjects is expected to be sufficient to identify 100 p-tau217 positive cases for the BeyeOMARKER + cohort, and to determine a reliable prevalence estimate of AD pathology in our eye-clinic population.

### Participants

Participants will be recruited from a clinic for comprehensive eye-care (i.e., Bergman clinics) located in an area of Amsterdam known for its socio-culturally and socio-economically diverse population. To be eligible to participate, a subject 1) must be ≥ 50 years of age, and 2) did not receive a formal dementia diagnosis. Individuals visiting the eye clinic based on solely the following reasons are excluded from participation: 1) a traumatic insult, 2) a superficial inflammatory eye disease, and 3) a condition in a structure surrounding the eye that is not directly involved in visual processing (e.g. the tear-ducts and eye muscles). Individuals who are eligible and express their interest in the BeyeOMARKER study will receive written and oral information and are invited to the eye clinic for informed consent procedures and a screening visit at the Bergman eye-clinic after the mandatory consideration time (i.e., one week after receiving the participant information form).

For enrolment in the BeyeOMARKER + cohort, results of the plasma p-tau217 measurement will be prospectively evaluated based on an *a priori* defined cut-off for plasma p-tau217 positivity, established in a large independent data-set of patients and controls from the Amsterdam Dementia Cohort [113]. Subsequently, all p-tau217 positive participants (*n* = 100) and a group of matched p-tau217 negative controls (ratio 2:1, *n* = 50) will be selected to be included in the BeyeOMARKER + cohort (*n* = 150). Matching will be based on age, sex and eye condition categorized into 1) anterior eye conditions, 2) posterior eye conditions, 3) refractive errors, and 4) unexplained visual impairment to allow identification of individuals with suspected PCA(Table S1). Selected participants who are eligible (e.g., based on safety criteria described in Text S1) to participate will receive additional written and oral information on the BeyeOMARKER + study and will be invited to the Amsterdam UMC (location VUmc) for informed consent procedures and additional assessments after the mandatory consideration time.

### Base clinical dataset for all BeyeOMARKER participants

#### Pre-specified blood-based AD biomarkers: screening for AD pathology

For each participant, at least one EDTA blood tube (6 mL) is collected. This will primarily be used for evaluating the plasma p-tau217 level and secondarily for assessing the levels of plasma Aβ40, Aβ42, GFAP, and NfL using the N4PE (Neurology 4-Plex E) assay [114]. The complete panel of plasma p-tau217, Aβ40, Aβ42, GFAP and NfL has demonstrated diagnostic and prognostic performance for AD and neurodegenerative diseases, and their combined use has the potential to further improve the diagnostic and prognostic performance of blood tests [12, 115–117]. Both assays will be performed using the Simoa HD-X automated platform in line with standard lab procedures and in accordance with pre-analytical handling recommendations [114].

#### Future blood-biomarkers and genetic analyses: the BeyeOMARKER biobank

For participants who provide consent for the BeyeOMARKER biobank, three additional 6 mL EDTA blood tubes will be collected for storage of plasma and whole-blood in the BeyeOMARKER biobank. This will serve to conduct future genetic and biomarker research into (risk factors for) AD and dementia, for instance by investigating newly emerging plasma biomarkers and by exploring genetic risk modifiers. For example, APOE4 carriership is a known genetic risk factor for AD but findings related to the visual system have been counterintuitive. First, compared to amnestic AD, the prevalence of APOE4-carriership is lower in visual-variant AD and associations appear weaker [74, 118]. Second, even though eye diseases like age-related macular degeneration [50, 119] and glaucoma [120, 121] are associated with increased AD risk, APOE4-carriership appears a protective factor for these eye conditions. The BeyeOMARKER biobank will enable a rapid response to developments in the field to further optimize biomarker-based diagnostic algorithms, and may provide more insight into genetic risk factors for AD and conditions of the visual system.

#### Sociodemographic and medical data collection

The collection of sociodemographic information serves to evaluate how representative our study sample is to the general population, and to investigate whether there are group-differences associated with sociodemographic factors that call for stratification and/or tailored interpretation of AD risk-estimates. Variables include sex, age, marital status, socio-economic status (SES), country of birth (age of immigration, if applicable) and country of birth of the parents and ancestors. Collection of country of birth is based on the updated guidelines provided by the Dutch central bureau of statistics (CBS) in 2022 [122]. SES is based on overall SES of the resident living community (information provided by the CBS), educational attainment [123, 124] and occupational attainment [125].

General and ophthalmological medical history will be collected to evaluate their associations with biomarker measurements and to investigate shared risk factors and pathological features between eye-disease and dementia. General medical history includes current diagnoses, medication use, relevant family history, and an assessment of cardiovascular risk factors (e.g. length and weight for body mass index, smoking, alcohol use, diabetes, blood pressure, treatment status [126]). Ophthalmological medical history includes presence of eye disorders, ophthalmological interventions and self-reported (functional) visual impairment with use of visual aids based on the Dutch EyeQ itembank [127].

#### Repeated cognitive screening and questionnaires

Cognitive screening will be performed using the Dutch or English version of the Montreal Cognitive Assessment (MoCA) standard or MoCA blind. The MoCA is a validated tool to screen for cognitive impairment and covers all cognitive domains (visuospatial function, executive function, language, memory and attention/processing speed [128]). The MoCA blind [129, 130] is similar to the standard MoCA but leaves out the vision-dependent subtasks making it suitable to administer to visually impaired participants. The MoCA blind also allows annual remote cognitive screening via telephone, which will be combined with online follow-up questionnaires to track medical and ophthalmological changes. Additional questionnaires including patient-centered outcomes (e.g. health, mobility, work-status, social environment and use of healthcare) and a web-based cognitive test (cCOG; [108]) can be incorporated from the ABOARD platform [109].

### Extended clinical dataset for the BeyeOMARKER + cohort

#### (Hyperspectral) Retinal imaging

In the current study, HS retinal imaging will be performed using the Optina Mydriatic Hyperspectral Retinal Camera (MHRC). Unlike conventional retinal cameras, the Optina MHRC contains an integrated light source that emits monochromatic light of different wavelengths onto the retinal surface. The camera images a 31° field-of-view of the retina and acquires 92 retinal images for successive monochromatic wavelengths in one second (5 nm increments across a visible to near-infrared spectral range of 450–905 nm). This way, a HS retinal scan provides a stack of monochromatic images containing both spatial and spectral information (i.e., each spatial locus has an associated reflectance across wavelengths). Parameters from these data-rich retinal images have been correlated to amyloid status (positive or negative) to build a ‘Retinal Deep Phenotyping’ model. This model incorporates phenotypic features that provides a probability of amyloid positivity [97, 98, 102–104]. Optina’s existing model will be used to predict the Aβ-PET and Tau-PET status of BeyeOMARKER participants.

Other imaging modalities that have been extensively reviewed [15, 16, 37–39] and are in line with a recommended minimum data set framework provided by experts in neuroscience, neurology, optometry and ophthalmology [16] are optical coherence tomography (OCT; Heidelberg spectralis), OCT-A (OCT-angiography; Zeiss plex elite 9000), and (blue autofluorescence) widefield fundus imaging (Optos). OCT provides structural information, such as the thickness of the retinal layers at the macular region and at the optic disc. The OCT-A yields vascular parameters, such as vessel density in the macular area and around the optical nerve head. In addition, a widefield fundus photo allows visualization of the far periphery of the retina (i.e., 200 degrees or 80% of the retinal surface), which has been shown to contain significant AD pathology as well [42]. Finally, blue autofluorescence imaging adds information on fluorescent properties of pigments in the retina, which is informative for various retinal disorders (e.g. age related macular degeneration, macular dystrophies) and potentially AD-related pathological changes [15, 131, 132]. Altogether, these imaging techniques could provide more insight into the eye-brain connection and in which of the parameters provided by a HS retinal scan contribute (the most) to the classification of AD biomarker status, particularly since HS imaging specifically for AD detection purposes has been validated in populations without eye conditions.

To ensure retinal image quality, participants first undergo pupil mydriasis achieved by administration of Tropicamide 0.5% drops into both eyes according to standard procedure ophthalmological clinical practice. If one eye is not suitable for retinal imaging, pupil mydriasis and subsequent scanning is performed on a single eye.

#### Structural MRI

Structural MRI will be performed to assess associations with our primary screening biomarkers (plasma p-tau217 and HS retinal scans) and to gain a deeper understanding of the interplay between conditions of the visual system, AD pathology and the down-stream effects of pathology (e.g. atrophy and white matter damage). Images are acquired on a 3T MR scanner at the Amsterdam UMC (location VUmc). To minimize participant burden we only include the following standard sequences: sagittal 3D T1, axial T2, Axial Susceptibility Weighted Image (SWI), Axial Diffusion Weighted Image (DWI) and Sagittal 3D Fluid-attenuated inversion recovery (FLAIR). These sequences are part of the standard diagnostic protocol for dementia at the Amsterdam UMC and provide neurodegenerative markers including cortical thickness, grey matter volume, white matter volume, and cerebrovascular outcomes such as white matter hyperintensities, lacunes and microbleeds.

#### Aβ-PET and tau-PET visual read and quantification

Aβ-PET and tau-PET are a validated reference standard to evaluate novel AD biomarkers [13]. Abnormality on both Aβ-PET and tau-PET is strongly associated with short-term subsequent cognitive decline [133] and, beyond binary classification, PET allows valuable insight into the extent and regional distribution of pathology [134, 135]. PET scans will be performed on a Siemens Whole-Body PET-CT-scanner (Biograph Vision Quadra) as this scanner provides excellent imaging results at lower tracer dosages. For the Aβ-PET scan acquisition, participants receive a single intravenous bolus injection of approximately 140 MBq [^18^F]florbetapir and undergo a static scan from 50 until 70 min post-injection. For the tau-PET scan, participants receive a single intravenous bolus of approximately 140 MBq [^18^F]flortaucipir and undergo a static scan from 80 until 100 min post-injection. Scanning procedures also include acquisition of a low-dose Computerized Tomography (CT) scan prior to the PET scan for attenuation and motion correction. After PET scan acquisition, the scans will be reconstructed into 4 × 5-min frames, corrected for movement when necessary, co-registered to the corresponding T1 MR image, and reoriented to remove head tilt. Visual reads will then be performed in correspondence with company guidelines for [^18^F]florbetapir (Amyvid) and [^18^F]flortaucipir (Tauvid) [136, 137]. Furthermore, semi-quantification will be performed by calculating standardized uptake value ratios (SUVR) to address our secondary study objectives [137–145].

#### Cognitive and cortical vision assessment

Cognitive and cortical vision assessment will be performed to assess the clinical effects of AD pathophysiological changes, to assess clinical trajectories in the BeyeOMARKER cohort and to the determine the presence of suspected PCA based on positive AD biomarkers and adherence to clinical criteria for PCA (i.e., based on cognitive and cortical vision tests) [73].

The comprehensive cognitive test battery (Table S3) covers all cognitive domains based on vision-dependent as well as non-vision-dependent tasks (with exception of the visuospatial domain, which includes the Visual Object and Space Perception Battery [VOSP] and is inherently vision dependent). Of note, given the expected cultural and educational diversity of the study population, a short 20-item version of the Naming Assessment in Multicultural Europa (NAME) task will be administered [146], which is less culture- and education-dependent compared to other naming tasks. Furthermore, most tasks are suitable administer and execute in English when appropriate (e.g., Rey-complex figure, digit-span task, trail making task, and the VOSP). Additional cortical vision tests (Table S4) will cover all basic visual perception and visual spatial processing domains based on tasks from the Cortical Vision Screening Test (CORVIST) and the self-report Colorado screening questionnaire for posterior cortical symptoms [147] as recommended by the Atypical AD Professional Interest Area of the Alzheimer’s association [148].

### Outcome measures

The performance of plasma p-tau217 and AI-based Aβ-status classification from the HS retinal scan will be evaluated for detecting AD pathophysiology and cognitive decline. First, it is essential to evaluate novel AD biomarkers against an extensively validated reference standard like PET [13]. Therefore, the primary pathophysiological outcome of interest is the visual read of the Aβ-PET and tau-PET scan to determine positivity for AD biomarkers. Visual examination will be performed by by a trained nuclear medicine physician in accordance with the company guidelines for [^18^F]florbetapir (Amyvid) and [^18^F]flortaucipir (Tauvid) [136, 137]. Second, the primary clinical outcome of interest is change on the modified preclinical Alzheimer cognitive composite 5 (mPACC5 [133, 149]) across a 21–24 month interval (i.e., timepoint T0 to T2). For the BeyeOMARKER study, the mPACC5 will be compiled as a vision-independent composite of the Rey Auditory verbal Learning test delayed recall (episodic memory), digit-span backward (executive function), animal fluency (semantic memory) and the MoCA blind (global cognition).

### Statistics

Statistical analyses will be performed using R studio. First, the performance of plasma p-tau217 and AI-based Aβ-status classification from the HS retinal scan will be determined based on logistic regression and Receiver operating characteristic (ROC) analysis for 1) presence of AD pathophysiology defined as a positive Aβ-PET and/or tau-PET visual read and 2) clinical decline defined as ≤ -1 versus > -1 standard deviation decline on the mPACC5). The logistic regression models will be performed including plasma p-tau217, the HS scan, and both methods combined to compare their performance to detect cognitive decline and AD pathophysiology. Models will be corrected for age and sex, and additionally for educational attainment when assessing cognitive outcomes. The ROC curve will be calculated using the predicted probabilities from the logistic regression models and sensitivity, specificity, accuracy, positive predictive value, negative predictive value and the Area Under the Curve (AUC) will be derived to assess the models’ discriminative power. Appropriate tests will be used to compare the performance between biomarkers (e.g., the DeLong test to compare AUCs).

In secondary analyses (Table 4), general linear and non-linear models will be explored to assess and compare the performance of p-tau217 and the retinal scan to predict down-stream effects of AD (e.g. MRI markers and cognitive and cortical vision outcomes). We will additionally compare MRI features and cognitive measures between the BeyeOMARKER cohort and an independent reference sample from the Amsterdam Dementia Cohort [113] to explore how comorbid eye-disease affects the neurobiological and clinical manifestations of AD. Since these outcomes may also be affected by other comorbid conditions (e.g. other neurological or psychiatric conditions), this will be evaluated in post-hoc assessments. Lastly, we aim to report the observed prevalence of plasma p-tau217 positivity in the BeyeOMARKER cohort and compare our findings with a memory clinic cohort and the general population, while also exploring the effect of demographic features (such as age, sex, SES and APOE genotype) using general linear models.

### Ethical considerations

#### General ethical considerations

The BeyeOMARKER study will be conducted in accordance with the Medical Research Involving Human Subjects Act (WMO) and according to the principles of the World Medical Association (WMA) Declaration of Helsinki, version 64 WMA General Assembly, Fortaleza October 2013. The study will be conducted in compliance with the protocol Clinical Trials Regulation No 536/2014 and with the principles of good clinical practice (GCP). Data and human material will be handled confidentially and in agreement with the Dutch Act on Implementation of the General Data Protection (GDPR) (in Dutch: algemene verordening gegevensbescherming; AVG).

The study has been reviewed and approved by the Medical Ethics Committee from the Amsterdam UMC. Adequate time, a week at minimum, will be given for the subject to consider his or her decision to participate in the study. Consent procedures will clarify that consent can be withdrawn at any stage, and research participants can refuse participation in any of the BeyeOMARKER study procedures at any time without consequence. Optional consent will be obtained with regard to sharing of data for countries outside the European Union. Consent procedures make it clear that data protection is either at an adequate level of data protection based on article 45 of Regulation (EU) 2016/679 (Adequacy decisions (europa.eu) (e.g. for Canada) or will be at the best possible level of confidence when other standards apply (e.g. for the United States).

#### Ethical considerations around biomarker disclosure

For all personal data, BeyeOMARKER follows a non-disclosure policy, meaning that one’s own personal data will never be automatically disclosed to the individual. However, participants may still learn their study results when the treating physician considers it clinically relevant and responsible to disclose a result or when legal requirements around personal data oblige the study to return personal data to the participant when this is requested.

A recent systematic review reported high interest in biomarker disclosure (72–81% for individuals involved in research and 50% in the general population) and no significant short-term psychological effects. Moreover, disclosure was generally considered actionable in terms of implementing lifestyle changes, seeking clinical trial participation and preparing for the future (e.g. financial, legal and living arrangements) [150]. However, the personal attitude towards biomarker disclosure and the consequent impact is highly personal and remains dependent on the clinical, personal and societal context. Furthermore, as the landscape around Alzheimer biomarkers and care will continue to change, so will the ethical considerations around biomarkers disclosure. In the BeyeOMARKER study we aim to further minimize the risk of negative impacts. First, the BeyeOMARKER study is initiated by a specialized memory clinic with longstanding experience at the forefront of innovative biomarker research, which has provided extensive experience with novel biomarker interpretation, disclosure and communication. Second, the BeyeOMARKER study implements a disclosure protocol in order to standardize procedures that ensure understanding and mitigate the impact of receiving information on one’s own AD biomarker status. With these strategies in place, participants are supported in making informed decisions concerning their own biomarker data.

## Results

The Medical Ethics Committee approved the BeyeOMARKER study in March 2024. We aim to implement the current protocol between April 2024 and March 2027 and are intending to seek additional funding for extended annual follow-up. Primary outcomes include the performance of plasma p-tau217 and HS retinal scanning for 1) Aβ-PET and tau-PET visual read as reference standard, and 2) cognitive change (Table 4).

## Discussion

The BeyeOMARKER study is a single-center prospective, observational, longitudinal cohort study that aims to evaluate both blood- and eye-based screening tools for early detection of AD in a cohort of patients from a clinic for comprehensive eye-care. First, the implementation of optimized multimodal screening outside of a specialized memory clinic setting has the potential to make early AD detection more accessible and cost-effective, thereby reducing the per-person cost for an AD diagnosis compared to existing tools [84]. This will aid in facilitating accessibility of early interventions that improve patient- and caregiver wellbeing [4–6], which will in turn reduce long-term care costs [151, 152]. Second, the multimodal dataset in a unique study population of eye patients could increase our understanding of the eye-brain connection and provide new routes for early intervention, potentially even for both classes of disease (i.e., brain and eye disease). Recently, the population attributable fraction (PAF) of vision impairment of dementia was estimated to be 1.8%, meaning that a proportion of these dementia cases could have been prevented by appropriate management of eye disorders [54]. Despite this seemingly low percentage, vision impairment is deemed an important factor to consider in life-course models of potentially modifiable dementia risk factors [54] given that 9 out of 10 cases of vision impairment are preventable or treatable by relatively simple and cost-effective interventions (e.g. corrective lenses or cataract surgery). The observed co-existence of visual and cognitive impairment and the availability of effective, yet underused, ophthalmological interventions suggest an important interplay between ophthalmological and memory clinic practice that could allow relatively easily obtainable health and quality of life benefits [52, 153].

### Complementary value of blood- and eye-based biomarkers

Thus far, blood- and eye-biomarkers have not been applied in a combined multimodal screening approach. Hence, the (extent of) added value of applying these biomarker modalities in conjunction remains a key question to be addressed in the BeyeOMARKER study. Multimodal biomarker approaches for AD are gaining traction to improve AD detection, prognosis, and monitoring. After all, AD is a complex disease with many pathophysiological contributors and each modality has its own strengths and limitations in capturing different aspects and stages of AD-related pathophysiological changes [13, 14, 154, 155]. Currently, several blood tests allow detection of AD-pathology with high accuracy, including the core pathophysiological hallmarks, as well as neurodegenerative and inflammatory markers [12]. However, the interpretation of blood-based biomarkers may be affected by variability due to interindividual differences in general systemic metabolism, or comorbidities (e.g., obesity, chronic kidney disease, cardiovascular conditions) and/or sociodemographic factors (e.g., sex, diversity in race or ethnicity) that potentially affect metabolic rates [156, 157]. In contrast, retinal imaging provides an accessible way to directly visualize the retinal component of the CNS, thereby offering a direct insight into molecular changes (e.g., protein depositions) and structural changes (e.g., neurodegenerative and vascular changes) [14, 107]. Interindividual differences in, and dynamic changes of, systemic metabolism will less likely impact structural retinal imaging parameters compared to dynamic blood-biomarker concentrations. However, retinal changes may occur in other (neurodegenerative) diseases and are less AD-specific [36, 39, 107] than markers of plasma p-tau. We therefore hypothesize that retinal markers should not be regarded as an alternative to blood-based biomarkers but rather that combining eye- and blood-based could have complementary value in detecting AD pathophysiology and cognitive decline.

### Future opportunities

The characterization of the BeyeOMARKER cohort provides multiple avenues for future research beyond the objectives outlined in this report. First, the field of blood-biomarkers is evolving rapidly and creating a biobank allows future assessment of novel and potentially better performing biomarkers. Secondly, questionnaires implemented in the online ABOARD platform [109] provides low burden collection of long-term functional outcomes in relation to AD(-related) blood-biomarkers or to eye disease and visual impairment. Third, multiple opportunities exist for AI-based classification of HS retinal scans. For example, it is thus far unclear which of the myriad of parameters provided by a HS retinal scan contribute (the most) to the classification of AD biomarker status, and whether these parameters are directly reflective of amyloid pathology or of other pathological processes like iron accumulation, mitochondrial dysfunction, or inflammation [98, 158, 159]. Furthermore, retinal depositions of tau are observed in glaucoma [160] as well as in AD [161, 162] and a recent study suggests spectral signature related to retinal tau *ex vivo* [163]. Currently, the question remains whether AI-driven classification of data-rich HS images could provide retinal-indices that 1) relate to tau-PET status or to a combination of Aβ-PET and tau-PET status, and 2) remain specific to AD in cases with a simultaneous eye disease affecting the retina. These developments, alongside the rapid developments of novel blood-based biomarkers, may provide novel multimodal screening approaches for optimized prognostication. Fourth, implementation of PCA screening tasks may give an estimate on the number of patients that present at the eye clinic with cortical (rather than ocular) vision complaints, indicative of early PCA [164]. Depending on the sample size, this subgroup is highly suitable to examine the role ophthalmological practice in identifying potential PCA cases and to further characterize the first symptoms and progression of these early PCA cases. Other future ambitions include the implementation of additional longitudinal follow-up for blood-based and eye-based assessment to study the dynamics of these markers and to assess the predictive value of changes over time.

### Challenges

Given the novelty and ambitious nature of the BeyeOMARKER study design, a number of challenges are anticipated. First, although screening for cognitive complaints in eye care settings has been proposed before [34, 46], little is known about the willingness of patients to undergo screening, or of eye care professionals to perform this screening. Recent literature suggests that out of 210 participants from a senior center, 194 (92.4%) would want to know their dementia risk based on retinal scanning, particularly to be able to plan for the future [165]. A supportive attitude towards cognitive screening was also reported for audiology services, but training of the audiologist and sufficient explanation was deemed important [166]. The latter finding points out the general challenge regarding investment of time and staff resources, and the degree of willingness to make these investments is currently unknown among ophthalmologists. Secondly, the targeted sample size of 700 participants is ambitious, particularly in currently under-represented socio-culturally and socio-economically diverse populations where enrollment barriers are relatively high [62, 167, 168]. Recruitment will be continuously monitored, and our criteria and recruitment strategies may be adapted throughout the study when deemed necessary. Alternatively, the BeyeOMARKER project will continue as planned but with reduced sample sizes. Third, additional study procedures for the BeyeOMARKER + cohort can be experienced as relatively burdensome. Even though the procedures are standard clinical procedures with known and acceptable risks, in this part of the study we may encounter reduced willingness to participate [169]. Therefore, we aim to minimize study burden where possible by scheduling visits at a familiar location (i.e., the eye clinic), implementation of home-based online questionnaires, providing flexibility in scheduling, providing a clear and accessible point of contact and ensuring understanding of the relevance and burdens of study procedures. The latter may be particularly relevant for the PET scan procedures as this is a known study enrolment barrier, especially in some previously underrepresented groups [170]. Therefore, the study team will follow recommendations on the communication regarding PET-scanning, such as efforts to improve understanding of the (minimal) risks of radiotracers by avoiding jargon, using visualization aids, providing understandable risk estimates and implementing active listening strategies [171]. Finally, challenges remain in cognitive assessment of participants with a visual impairment or culturally diverse background, particularly as the solutions can be counteracting. For example, tasks adapted for participants with a visual impairment are often more language-dependent, while tasks adapted to culturally and linguistically diverse populations are often more vision-dependent. Any potential language- or vision-dependent bias in cognitive testing will be documented and will be taken into account through sensitivity analyses when evaluating the clinical outcomes. We will report on our findings with regard to the performance of our clinical measures to inform future investigations.

## Conclusions

The BeyeOMARKER study will provide a well-characterized cohort to 1) investigate the feasibility of early AD detection based on blood- and eye-based biomarkers in alternative screening settings, and 2) improve our understanding of the eye-brain connection. Findings, future opportunities, challenges and limitations of the BeyeOMARKER study will be integrated into a roadmap for large-scale implementation of early AD detection, which will aid towards building an efficient and inclusive infrastructures to detect individuals at risk of AD and allow intervention to those who need it.

### Supplementary Information

Supplementary Material 1.

## Data Availability

No datasets were generated or analysed during the current study.

## Abbreviations

ABOARD: A Personalized Medicine Approach for Alzheimer's Disease
AD: Alzheimer's disease
Aβ: Amyloid beta
AI: Artificial Intelligence
APOE: Apolipoprotein E
CBS: Dutch central bureau of statistics
CORVIST: Cortical Vision Screening Test
EU: European Union
GCP: Good Clinical Practice
GDPR: General Data Protection (GDPR) (in Dutch: algemene verordening gegevensbescherming (AVG))
HR: Hazard Ratio
HS: Hyperspectral
MHRC: Mydriatic Hyperspectral Retinal Camera
MoCA: Montreal Cognitive Assessment
MRI: Magnetic Resonance Imaging
N4PE assay: Neurology 4-Plex E assay
OCT: Optical coherence tomography
OCT-A: OCT-Angiography
PAF: Population Attributable Fraction
PCA: Posterior Cortical Atrophy
PET: Positron Emission Tomography
p-tau: Phosphorylated tau
RR: Relative Risk
SES: Socioeconomic status
Simoa: Single-molecule assay
TMT: Trail Making Task
VI: Visual impairment
VOSP: Visual Object and Space Perception Battery
VUmc: VU University Medical Center
WMA: World Medical Association
WMO: Medical Research Involving Human Subjects Act
