# Supplementary material for: Rationale and design of the BeyeOMARKER study: prospective evaluation of blood- and eye-based biomarkers for early detection of Alzheimer’s disease pathology in the eye clinic
Source: Alzheimers Res Ther. 2024 Aug 21;16:190. doi: 10.1186/s13195-024-01545-1 (PMC11340081; doi:10.1186/s13195-024-01545-1)
Supplement: Supplementary file 1 — Supplementary Material 1. [file 13195_2024_1545_MOESM1_ESM.docx]

# Supplementary material

**Table S1 – Categorization of eye conditions.**

| **Eye condition category** | **Description** |
| --- | --- |
| 1. **Anterior** | Conditions affecting the anterior segment of the eye (e.g. cornea, iris, ciliary body, and lens) including cataract, uveitis and corneal disease. |
| 1. **Posterior** | Conditions affecting the posterior segment of the eye (e.g. vitreous humor, retina, choroid, and optic nerve) including diabetic retinopathy, macular degeneration and glaucoma. |
| 1. **Refractive errors** | Astigmatism, hyperopia, myopia, presbyopia. |
| 1. **Unexplained visual conditions** | Loss of visual acuity, seeing dots and flashes, acquired trouble in reading and writing. |

## Table S2 – The BeyeOMARKER and BeyeOMARKER+ study procedures.

| **BeyeOMARKER cohort**  **(n=700)** | **BeyeOMARKER+ cohort***  **(n=150)** |  |
| --- | --- | --- |
|  | **Screening visit** | |
| Plasma p-tau217 | ✔ | ✔ |
| Plasma Aβ40, Aβ42, GFAP, NfL | ✔ | ✔ |
| Genetic risk factors | ✔* | ✔* |
| Other AD biomarkers | ✔* | ✔* |
| Sociodemographic variables | ✔ | ✔ |
| Medical history | ✔ | ✔ |
| Cognitive screening | ✔ | ✔ |
|  | **On-site T0**  ±3 (max. 6) months after screening | |
| Cognitive assessment |  | ✔ |
| Cortical vision assessment |  | ✔ |
| Structural MRI |  | ✔ |
| (Hyperspectral) retinal imaging |  | ✔ |
| Aβ-PET and tau-PET |  | ✔ |
|  | **Remote T1**  9-12 months after screening | |
| Cognitive screening | ✔ | ✔ |
| Questionnaires | ✔ | ✔ |
|  | **Remote T2**  9-12 months after T1 | |
| Cognitive screening | ✔ | ✔ |
| Questionnaires | ✔ | ✔ |
|  | **On-site T2**  21-24 months after T0 | |
| Cognitive assessment |  | ✔ |
| Cortical vision assessment |  | ✔ |
| Structural MRI |  | ✔ |

Overview of the study procedures for the complete BeyeOMARKER cohort (blue) and specific study procedures for the BeyeOMARKER+ cohort (green). *Abbreviations*: Aβ = amyloid β, GFAP = glial fibrillary acidic protein, NfL = neurofilament light, MRI = magnetic resonance imaging, PET = Positron Emission Tomography, HS = hyperspectral, *****only applicable if the required optional consent has been provided.

## Table S3 – Neuropsychological test battery.

| **Domain** | **Cognitive test** | **Vision-dependent?** |
| --- | --- | --- |
| **Global** | MoCA | Yes/No |
| **Memory** | Rey Auditory Verbal learning test | No |
|  | Rey-complex figure memory condition | Yes |
| **Executive function** | Digit span backward | No |
|  | Letter fluency | No |
|  | TMT-B | Yes |
| **Language/ semantic memory** | Fluency animals | No |
|  | NAME20 | Yes |
| **Attention and speed** | Digit span forward | No |
|  | TMT-A | Yes |
| **Visuospatial function** | Rey complex figure copy condition | Yes |
|  | VOSP dot counting, fragmented letters and number location | Yes |

*Abbreviations*: MoCA = Montreal Cognitive Assessment, NAME = Naming Assessment in Multicultural Europe, TMT = Trail Making Task, VOSP = Visual Object and Space Perception Battery.

## Table S4 – Cortical vision assessment.

| **Cortical vision feature** | **Test** | **Vision-dependent?** |
| --- | --- | --- |
| **Early visual processing deficits** | CORVIST shape discrimination | Yes |
|  | CORVIST size discrimination | Yes |
|  | CORVIST shape detection test | Yes |
|  | CORVIST symbol acuity | Yes |
| **Central achromatopsia** | CORVIST hue discrimination | Yes |
| **Visual crowding** | CORVIST crowding test | Yes |
|  | Colorado questionnaire | No |
| **Simultanagnosia** | Poppelreuter Ghent Overlapping figure | Yes |
|  | Navon figure | Yes |
|  | Colorado questionnaire | No |
| **Object perception deficit** | **VOSP fragmented letters* | Yes |
|  | CORVIST unusual/usual views | Yes |
|  | CORVIST fragmented numbers | Yes |
|  | Colorado questionnaire | No |
| **Space Perception Deficit** | **Rey complex figure copy condition* | Yes |
|  | **VOSP number location* | Yes |
|  | **VOSP dot counting* | Yes |
|  | CORVIST dot counting | Yes |
|  | Colorado questionnaire | No |
| **Constructional dyspraxia** | **Rey complex figure copy condition* | Yes |
| **Agraphia** | Promp to spontaneously write one sentence | Yes |
| **Alexia** | Paragraph reading | Yes |
|  | CORVIST word reading | Yes |
|  | Colorado questionnaire | No |
| **Acalculia** | Verbal calculations from prepared script | No |
|  | Colorado questionnaire | No |
| **Apperceptive prosopagnosia** | CORVIST face perception 1 & 2 | Yes |
|  | Colorado questionnaire | No |
| **Left-right disorientation** | Ask patient to point to left and/or right side of body of self and examiner | No |
|  | Colorado questionnaire | No |
| **Finger agnosia** | Not assessed. | NA |
| **Environmental agnosia** | Self-report for familiar places, unfamiliar places and inside the house. | No |
| **Other dominant parietal dysfunction** | **Digit span forward and backward* | No |
| **Homonymous Hemianopia** | Collected as part of medical history. | No |

*Abbreviations*: CORVIST = Cortical Vision Screening Test, VOSP = Visual Object and Space Perception Battery**,** *Test is covered in cognitive testing battery table S3.

## Text S1: Additional exclusion criteria for the BeyeOMARKER+ cohort

A potential subject who meets any of the following criteria will be excluded from participation in the BeyeOMARKER+ cohort:

- The participant is unable to complete the study procedures as judged by the investigator
- Pupil dilation required for the retinal scans is inadequate or contraindicated
- Both eyes are affected by a condition that severely impacts hyperspectral retinal scan acquisition. For example, due to insufficient light transmittance (dense cataract, severe vitreous floaters) or uncontrolled eye movements (nystagmus).
- Magnetic Resonance Imaging (MRI) is contraindicated
- The participant has known hypersensitivities to the active ingredients of [^18^F]florbetapir and [^18^F]flortaucipir, or relevant history of severe drug allergy of hypersensitivity.
- The participant already exceeds the total yearly radiation exposure limit for clinical research of 11.3 mSv for females and 15.3 mSv for males.
- The participant has a known history of structural brain abnormalities that are likely to interfere with the interpretation of the position emission tomography (PET) scan (e.g. major stroke or mass)
- Women of childbearing potential (WOCBP) according to the Clinical Trials Coordination Group (CTFG) recommendations are excluded if they are not refraining from sexual activity or are not using reliable methods for contraception described in the CTFG recommendations.
- If WOCBP are included, they must confirm not to be pregnant or breast feeding prior to the PET scan.
